# A multi-center, double-blind, randomized, placebo-controlled trial protocol to assess Traumeel injection vs dexamethasone injection in rotator cuff syndrome: the TRAumeel in ROtator cuff syndrome (TRARO) study protocol

**DOI:** 10.1186/s12891-015-0471-z

**Published:** 2015-02-04

**Authors:** Luc Vanden Bossche, Guy Vanderstraeten

**Affiliations:** Physical and Rehabilitation Medicine, Sportsmedicine, Ghent University Hospital, De Pintelaan 185, 9000 Ghent, Belgium; Department of Rehabilitation Sciences and Physical Therapy, Faculty of Medicine and Health Sciences, Ghent University Hospital, De Pintelaan 185, 9000 Ghent, Belgium

**Keywords:** Rotator cuff syndrome, Shoulder, Bursitis, Traumeel, Dexamethasone, Corticosteroids, Ultrasound, Injections, Pain

## Abstract

**Background:**

Shoulder pain is a common musculoskeletal symptom with a wide range of potential causes; however, the majority of conditions can be managed with conservative treatment. The aim of this study is to assess the efficacy and safety of Traumeel injections versus corticosteroid injections and placebo in the treatment of rotator cuff syndrome and bursitis and expand the current evidence base for the conservative treatment of rotator cuff syndrome.

**Methods/Design:**

This is a multi-center, randomized, double-blind, 16-week, three-arm, parallel-group, active- and placebo-controlled trial to assess the efficacy and safety of Traumeel 2 ml injection versus dexamethasone 8 mg injection versus placebo (saline solution). Patients will be randomly allocated to Traumeel, dexamethasone or placebo in a 2:2:1 randomization. After 1 week screening, patients will receive 3 injections at weekly intervals (days 1, 8 and 15) with additional follow-up assessments on day 22, a telephone consultation in week 9 and a final visit at week 15. Male and female patients aged 40 to 65 years, inclusive, will be recruited if they have acute episodes of chronic rotator cuff syndrome and/or bursitis. Patients with calcifications in the shoulder joint or a complete rotator cuff tear will be excluded. At least 160 patients will be recruited. All subacromial injections will be performed under ultrasound guidance utilizing a common technique. The only rescue medication permitted will be paracetamol (acetaminophen), with usage recorded. The primary endpoint is change from baseline in abduction-rotation pain visual analog scale (0–100 mm scale, 0 corresponds to no pain and 100 to extreme pain) at day 22 (Traumeel injections versus dexamethasone injections) for active external rotation. Secondary efficacy parameters include range of motion, disability of arm, shoulder, hand score and patient’s/investigator’s global assessment. Clinical efficacy will be assessed as non-inferiority of Traumeel with respect to dexamethasone regarding the primary efficacy parameter.

**Discussion:**

It is hoped that the results of this trial will expand the treatment options and evidence base available for the management of rotator cuff disease.

**Trial registration:**

ClinicalTrials.gov: NCT01702233. EudraCT number: 2012-003393-12.

## Background

Shoulder pain is the third most common musculoskeletal symptom encountered in medical practice, after back and neck pain [1]. It accounts for almost 3 million patient visits each year in the USA [2,3]. A wide range of potential pathoanatomic entities, from simple sprains to massive rotator cuff tears, can give rise to shoulder pain [2].

The majority of these conditions can be managed with conservative treatment [2,4], and conservative therapy can include a number of novel treatments [5]. Rotator cuff dysfunction is a particularly important entity because it occurs frequently, and indicative with complete tear, may necessitate surgical treatment [2,6,7].

The shoulder has the greatest range of motion (ROM) of any joint in the human body. Size mismatch between the smaller glenoid and larger humeral head creates a risk of instability. Stability is provided both statically by the capsule and labrum, and dynamically by the rotator cuff musculature. Dysfunction of any of these structures can lead to pain, weakness, and instability.

The rotator cuff is a musculo-tendinous confluence of four muscles that initiate shoulder motion and maintain the normal relationship between the articular surfaces. The supraspinatus muscle provides abduction, the infraspinatus and teres minor muscles provide external rotation, and the subscapularis muscle provides internal rotation. In addition, the muscles of the rotator cuff balance the forces of other shoulder muscles, most importantly the deltoid muscle. Contraction of the deltoid muscle in the absence of supraspinatus function leads to superior translocation of the humeral head, making wide abduction difficult.

Non-operative treatment for shoulder pain due to rotator cuff impingement and tears generally includes appropriate physical therapy, anti-inflammatory medication, corticosteroid injections, and other approaches. Meta-analyses of trials of subacromial injection of corticosteroids for rotator cuff disease have shown a beneficial effect over placebo, while evidence for other interventions is lacking [6,8,9]. The importance of the accuracy of injecting the subacromial bursa with corticosteroids was highlighted by a study by Henkus *et al*. [10] showing that despite the confidence of physicians, without guidance many subacromial injections hit surrounding structures. However, only injection directly into the subacromial bursa resulted in significant pain relief and increase in functional scores. Marder *et al*. [11] further supported these findings and found that the rate of accuracy varied with route of injection, and anterior and lateral routes are more accurate than the posterior route. Due to potential variance in accuracy of subacromial injection between physicians, ultrasound-guided injections utilizing a common method have been used for this study.

Traumeel (Tr14) injection solution is a combination formula of 12 botanical and 2 mineral substances with demonstrated anti-inflammatory, anti-edematous, anti-exudative properties. The exact mechanism of action of Tr14 injection solution is still to be fully understood. Various cellular and biochemical pathways appear to be modulated by the ingredients. It has been suggested that Tr14 injection solution does not inhibit cyclo-oxygenase (COX) or lipoxygenase enzyme pathways, as is the case with non-steroidal anti-inflammatory drugs (NSAIDs) [12]. In the rat model of blood-induced inflammation, Tr14 injection solution significantly reduced hind paw induced-edema and decreased IL-6 production. The authors suggested that Tr14 injection solution seems to act by speeding up the healing process instead of blocking the development of edema from the beginning [13]. Additional basic research is currently underway to further elucidate Tr14 injection solution’s mechanism of action.

Tr14 injection solution has been shown to be effective for hemarthrosis of the knee [14], epicondylitis [15], and various musculoskeletal injuries [16]. However, no large study with Tr14 injection solution has been performed so far in patients with rotator cuff syndrome and bursitis. Thus, the aim of this study is to assess the efficacy and safety of Tr14 injection solution injections versus corticosteroid injections in the treatment of rotator cuff syndrome and bursitis and expand the current evidence base for the conservative treatment of the rotator cuff syndrome. It is hoped that the results of this trial will expand the treatment options available to clinicians for the treatment of rotator cuff syndrome, allowing greater patient choice.

## Methods/Design

**Figure 1:**
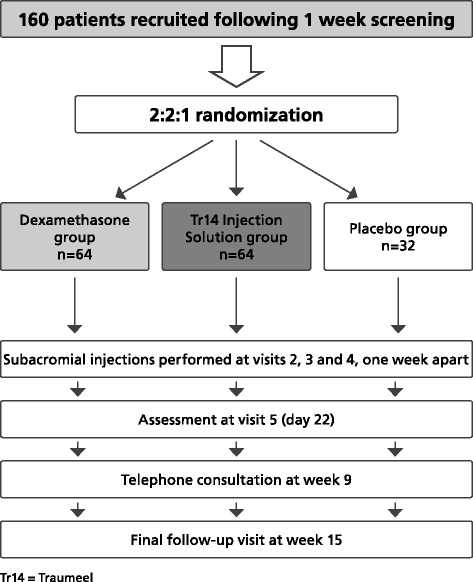
**Patient flow through the study.**

This will be a multi-center, randomized, double-blind, 16-week, three-arm, parallel-group, active- and placebo-controlled trial to assess the efficacy and safety of Tr14 injection solution 2 ml injection versus dexamethasone 8 mg injection versus placebo (Figure 1).

The objective of this study is to evaluate reduction of pain and improvement of functional motion parameters in patients with rotator cuff syndrome and bursitis treated with Tr14 injection solution injections versus corticosteroid injections and versus placebo.

Patients will be randomly allocated to Tr14 injection solution, dexamethasone or placebo in a 2:2:1 randomization. The randomization will be stratified by site. Randomization codes will be generated by a statistician not involved in the study from an algorithm based on the PROC PLAN procedure of SAS, Version 9.1.3. Sealed envelopes containing the individual codes will be sent to the centers and to the study sponsor for the purposes of assigning kits to patients and the managing of adverse events.

All study personnel and patients will be blinded to the treatment being used during the study. The investigator will keep the treatment code envelopes throughout the course of the study and must not break the code without valid reason, e.g. in the case of emergency, should stopping the blinded medication be considered insufficient to manage the individual patient. All study medication will be supplied in 2 ml vials ready for injection, and packaging and labeling will be carried out in accordance with the requirements of Annex 13 of the Good Manufacturing Practice (GMP) guidelines, International Conference on Harmonization (ICH) Good Clinical Practice (GCP) requirements, sponsor approved standard operating procedures, the European Union (EU) Clinical Trial Directive and all applicable local laws.

The study includes 2 sites in Belgium, 4 in Germany, and 4 in Spain, all based in outpatient clinics. The study has been approved by Competent Authorities in all three countries (Federal Agency for Medicines and Health Products in Belgium, Bundesinstitut für Arzneimittel and Medizinprodukte in Germany and the Agencia Espaňola del Medicamento in Spain), and has received ethical approval from relevant bodies in each country (Commissie voor Medische Ethiek Universitair Zirkenhuis Ghent in Belgium, Ethikkommission der Ärztekammer Hamburg in Germany and Comité Ético de Investigación Clinica Complejo Hospitalario de Toledo in Spain).

The study will be conducted in compliance with the ethical principles of the Declaration of Helsinki and its amendments as adopted by the 59^th^ World Medical Assembly (WMA) General Assembly, Seoul, October 2008; the principles of the GCP provided in the ICH Harmonised Tripartite Guidelines for GCP 1996; and all applicable national laws and regulations.

### Participants

Male and female patients aged 40 to 65 years, inclusive, will be recruited if they have acute episodes of chronic rotator cuff syndrome and/or bursitis: tendinopathy of the supraspinatus tendon, bursitis, or partial degenerative tears of the supraspinatus and/or infraspinatus tendon (differentiation by ultrasonography). They must be willing and able to understand and sign an approved informed consent form. Female patients must not be pregnant (as proven by negative pregnancy test before first study drug administration) or breast-feeding. Females of childbearing potential (including those less than one year post-menopausal) must agree to maintain reliable birth control throughout the study, i.e. an established use of oral, injected or implanted hormonal contraception, female sterilization by hysterectomy, bilateral oophorectomy, or bilateral tubal exeresis, intrauterine device (IUD) or coil or barrier method (e.g. diaphragm, cervical/vault cap) plus spermicidal cream/gel.

Potential study patients will be excluded if one of the following exclusion criteria is present: calcifications in shoulder joint; complete rotator cuff tears; treatment with NSAIDs (previous treatment with NSAIDs is allowed, with a wash-out period of 1 week; paracetamol [acetaminophen] can be taken until 48 hours before baseline visit); corticosteroid therapy by mouth or by injection within the previous 3 months prior to screening; any contraindication for corticosteroid therapy; physical therapy, acupuncture, transcutaneous electrical nerve stimulation (TENS) and shock-wave therapy (within 30 days prior to screening); treatment with anticoagulants (except low-dose aspirin); diabetic patients including borderline cases (glycosylated fraction of hemoglobin [HbA_1c_] >7.0% at screening); clinically significant shoulder joint deformities; major injury, including sports-related injury, to the shoulder within the past year; significant osteoarthritis of the shoulder; cervical spine disorder (that could confound the clinical assessment) that has been symptomatic and required active treatment within the past 3 months before screening; any active musculoskeletal disease that could confound the diagnosis/evaluation of the painful shoulder, any neurological etiology of the pain, or any acute infection of the shoulder joint; any major surgery, arthroplasty, or arthroscopy in the signal shoulder within 6 months of screening or planned surgery within the duration of the study; prior history of any malignancy (with the exception of basal cell carcinoma) treated less than 2 years ago; patients with rheumatic polymyalgia; known or suspected allergies against one or any particular ingredients of any of the study preparations; presence of serious gastrointestinal, renal, hepatic, pulmonary, cardiovascular, neurological disease or other known systemic disease (like leukemia, tuberculosis, immune mediated diseases, multiple sclerosis, Acquired Immuno Deficiency Syndrome, Human Immunodeficiency Virus-infections or other chronic virus infections) that might interfere with the outcome of the study or the patient’s ability to comply with study requirements; presence of infections and/or skin diseases in the area of the injection site (including psoriasis); clinically significant abnormal laboratory values (as judged of the investigator) at the screening visit; consumption of any investigational product within 1 month prior to the screening visit; and patients who are likely to be non-compliant or uncooperative during the study, as judged by the investigator.

### Recruitment

The recruitment phase will be completed when 160 patients have been enrolled, and the duration of this recruitment period will be about 12 months. Patients will be recruited from the existent patient pools at the trial centers. They will be outpatients attending for a scheduled visit who would then be asked about their willingness to be included in a clinical trial.

### Interventions

All interventions will be provided in 2 ml vials, identical in appearance. Traumeel® injection solution (Biologische Heilmittel Heel GmbH) is officially classified as a homeopathic medicinal product [17]. Tr14 injection solution is a formulation of 12 botanical and 2 mineral substances. The quantities of each component are shown in Table 1. Dexamethasone 8 mg will be provided as Fortecortin® (Merck Pharma GmbH) and placebo will be saline solution in 2 ml vials.

**Table 1:** **Components of Tr14 injection solution**

**Source of extract**	**Quantity per 2 mL injection solution**
*Achillea millefolium* (milfoil)	0.002 μL
*Aconitum napellus* (monkshood)	0.012 μL
*Arnica montana* (mountain arnica)	0.02 μL
*Atropa belladonna* (deadly nightshade)	0.02 μL
*Bellis perennis* (daisy)	0.01 μL
*Calendula officinalis* (calendula)	0.02 μL
*Matricaria recutita* (chamomile)	0.002 μL
*Echinacea angustifolia* (narrow-leaved cone flower)	0.005 μL
*Echinacea purpurea* (purple cone flower)	0.005 μL
*Hamamelis virginiana* (witch hazel)	0.02 μL
Calcium sulphide (otherwise: Hepar sulfuris)	0.000002 μL
*Hypericum perforatum* (St John’s wort)	0.006 μL
Mercurico-amidonitrate (otherwise: *Mercurius solubilis Hahnemanni*)	0.000001 μL
*Symphytum officinale* (comfrey)	0.000002 μL
Excipients	0.9% saline solution

Tr14 = Traumeel.

Participants will receive 3 injections at weekly intervals on visits 2, 3 and 4. All investigators were trained in ultrasound-guided injection technique at an investigator’s meeting in Ghent, Belgium, to ensure consistency of administration. The patient is placed in a supine position to reduce any risk of syncope during the procedure. Ultrasound guidance using a sagittal view (7 till 14 MHz) obtained over the lateral edge of the shoulder is used to guide the injection into the subacromial bursa with the ultrasound probe used in a long-axis lateral view. A narrow gauge needle should be used (22–30 gauge). The needle is advanced until its tip penetrates the bursal cavity. Images are taken before and after injection, showing distension of the bursa following injection of the product. For the first 48 hours after injection, the patient is allowed to continue all routine activities of daily living, but is advised not to overuse the treated shoulder.

### Concomitant care

Previous treatment with NSAIDs, analgesics, and COX type 2 (COX-2) inhibitors is allowed, with a washout period of 1 week before baseline; paracetamol can be taken until 48 hours before baseline visit. This also includes all kinds of applications, i.e. topical, oral or parenteral. Patients have to be instructed that for the duration of the study they must not take any pain relief medication other than paracetamol (which will be used as rescue medication and can be taken during the study except 48 hours before the study visits). No chondroprotective medication is allowed (e.g. among others, glucosamine, chondroitin sulfate, hyaluronic acid, diacerein, native collagen and so-called USA-300 preparation).

Treatment with anticoagulants is not permitted during the study. Low-dose (70–100 mg/d) aspirin for anti-thrombotic therapy is permitted if doses are stable for the month prior to screening and remain stable throughout the study period. Also, treatment with corticosteroid injections or intake of oral corticosteroids in the 3 months prior to the study or during the study is not permitted.

Physiotherapy is forbidden within 30 days prior to screening, but will be allowed as non-drug rescue treatment from Day 23 until Week 15. Acupuncture, TENS and shock-wave therapy are also forbidden within 30 days prior to screening and during the course of the study until week 15.

After screening, only paracetamol (500 mg when necessary) is permitted during the study as rescue medication for pain relief. At screening, after ensuring patient eligibility, paracetamol rescue medication and a patient diary will be provided to the patients. Patients will be instructed to document the paracetamol consumption every day and to bring the diary to the site at each visit, where paracetamol usage will be documented. Paracetamol consumption is limited to 2000 mg (4 tablets) per day. Patients are instructed that they must not take paracetamol within the 48 hours prior to a study visit.

### Criteria for withdrawal of patient from study

Study completion or discontinuation will be documented with the reason for any discontinuation. Reasons for a patient discontinuing participation in the study include:

- Inefficacy of the study therapy:
  - Increase of visual analog scale (VAS) by at least 30 mm in comparison to baseline during 2 consecutive visits
  - Any other medical condition requiring – in the opinion of the investigator – a change of the therapy for the baseline condition
- Occurrence of a medical condition requiring use of prohibited medications (NSAIDs, analgesics other than paracetamol, COX-2 inhibitors, chondroprotective medications, anticoagulants other than low-dose aspirin or corticosteroids other than study therapy)
- Medical condition affecting assessment of the primary endpoint (e.g. any injuries or conditions causing shoulder pain or requiring analgesic treatment)
- Medical conditions affecting patient safety if participation with the study therapy is continued: conditions and adverse events (AEs) causing safety concerns with intra-articular steroids therapy or with injection to the shoulder joint area OR any other AE or condition that – in the opinion of the investigator – endangers patient safety if the participation in the study is continued
- Withdrawal of consent
- Lost to follow-up
- Death.

In case of an AE, the patient is to be followed up until resolution of the AE. Patients who discontinue prematurely from the study will not be replaced.

### Adherence to protocol

Protocol adherence will be documented and judged by patient reporting (diary) and attendance at clinic visits according to schedule.

### Outcomes

The primary endpoint is change from baseline in abduction-rotation pain VAS (0–100 mm scale, 0 corresponds to no pain and 100 to extreme pain) at visit 5 (day 22) (Tr14 injection solution injections versus dexamethasone injections) for active external rotation. The abduction-rotation will be done with an internal rotation and external rotation and both actively and passively. However, the primary parameter is active rotation abduction with external rotation and only for this movement the VAS will be measured. During the shoulder examination, the active external abduction rotation must be the first movement during evaluation for pain VAS determination.

Secondary efficacy parameters include ROM, disability of arm, shoulder, hand (DASH) score and patient’s/investigator’s global assessment. For ROM the following movements will be analyzed [18,19]:

- Abduction rotation (active external, active internal, passive external, passive internal) measured by goniometry.
- Hand-back range and hand-neck range both measured in cm.
- Jobe (also known as ‘empty can’) test with measurement of pain and weakness (positive/negative). This will be examined as active movement.
- Painful arc after visit 4 (last injection) with measurement of pain (positive/negative). This will be examined as active movement.

Safety parameters include local tolerability, laboratory monitoring, vital signs and AEs. AEs will be standardized for terminology and classification, using Medical Dictionary for Regulatory Activities (MedDRA) (the latest available version will be used). Concomitant medications will be classified by site of action and therapeutic and clinical characteristics using the World Health Organization (WHO) DRUG dictionary (the latest available version will be used).

### Participant timeline

A schedule of study procedures and events is provided in Table 2.

**Table 2:** **Schedule of study procedures and events**

	**Visit 1 Screening (max. –7 days)**	**Visit 2 Baseline Day 1**	**Visit 3 Day 8 ± 1 day**	**Visit 4 Day 15 ± 1 day**	**Visit 5 Day 22 ± 1 day**	**Telephone Week 9 ± 3 days**	**Visit 7 Week 15 ± 3 days**
Informed consent	X						
Inclusion/exclusion review	X	X					
Body weight and height	X						
Physical examination	X						X
Vital signs	X	X	X	X	X		X
Medical history	X*						
Randomization		X					
Shoulder ultrasonography	X	X****	X****	X****			
Urine for pregnancy test	X	X					
Clinical laboratory tests	X				X		
Telephone visit						X	
Shoulder examination including***							
○ VAS score	X	X	X	X	X		X
○ DASH score							
○ Range of motion*****							
○ Jobe/painful arc test							
Patient’s global assessment					X		X
Investigator’s global assessment					X		X
Shoulder injections		X	X	X			
Previous and concomitant treatments*	X*	X	X	X	X	X	X
Rescue medication dispensation	X	X	X	X	X		
Patient diary dispensation	X						
Rescue medication consumption**		X	X	X	X		X
Patient diary collection							X
Study drug accountability		X	X	X	X		X
AEs	X	X	X	X	X	X	X

*Patients are to be instructed to discontinue their current pain medication (NSAIDs, analgesics, COX-2 inhibitors) one week prior to baseline visit. No chondroprotective medication is allowed (e.g., among others, glucosamine, chondroitin sulfate, hyaluronic acid, diacerein, native collagen and so-called USA-300 preparation).

**The usage of study rescue medication is generally not allowed within 48 hours before a study visit. At each visit, the patient has to bring the diary with documentation of daily consumption to the site.

***Bilateral shoulder examination at screening (VAS in target shoulder only).

****Ultrasound-guidance of subacromial periarticular study drug injections.

*****Range of motion includes abduction rotation (active external, active internal, passive external, passive internal) measured by goniometry and hand-back range and hand-neck range both measured in cm. The active external abduction rotation must be the first movement during shoulder examination for pain VAS determination.

AE = adverse event; COX-2 = cyclo-oxygenase type 2 inhibitors; DASH = Disability of arm, shoulder, hand; NSAID = non-steroidal anti-inflammatory drug; VAS = visual analog scale.

### Statistical methods

Clinical efficacy will be assessed as non-inferiority of Tr14 injection solution with respect to dexamethasone regarding the primary efficacy parameter. A one-sided test of non-inferiority of Tr14 injection solution with respect to dexamethasone at level 0.025 will be computed using an analysis of covariance (ANCOVA) model with treatment group and center as qualitative factors and the baseline value of the abduction rotation pain VAS for active external rotation as a covariate.

All continuous efficacy parameters will be analyzed by suitable analysis of ANCOVA models, whereas the dichotomous Jobe and painful arc test data will undergo suitable logistic regression model analyses. The ordered categorical responses of the patients’ and examiners’ global assessment will be evaluated by Cochran-Mantel-Haenszel (CMH) tests that account for stratification by center. Clinical safety will be addressed by assessing AEs, physical examinations, laboratory assessments, and vital signs results in a descriptive manner. All statistical analyses in this study will be exploratory in nature.

Analyses will be based on the safety analysis, full analysis, and the per-protocol sets. The summaries of the efficacy parameters, the statistical analyses of the primary efficacy variable, and the statistical analyses of the secondary efficacy variables will be performed on the per-protocol set. These summaries and analyses will be supported by corresponding summaries and exploratory statistical analyses performed on the full analysis set. Missing values for all efficacy parameters will be imputed by the last observation carried forward (LOCF) approach. All statistical tests will be supported by presenting estimates and 95% confidence intervals for the respective treatment effects and differences between the treatment groups. These estimates and confidence intervals will be based on the respective statistical models used for the analysis, shown in Table 3.

**Table 3:** **Statistical analyses to be performed**

ANCOVA with treatment group and center as qualitative factors and baseline value as covariate	Applicable for analyses at visits 5 and 7 for both treatment comparisons Tr14 injection solution against the comparators dexamethasone and placebo, respectively
	• Change from baseline in abduction-rotation pain VAS for active external rotation
	• Changes from baseline in ROM for abduction rotation as goniometry in degrees (active external, active internal, passive external, passive internal rotation)
	• Changes from baseline in ROM for hand-back range and hand-neck range as distance measurement in cm
	• Changes in DASH
Repeated measurements ANCOVA with treatment group, center, visit and treatment-by-visit interaction as qualitative factors and baseline value as covariate	Visits 5 and 7 included in analysis; both treatment comparisons Tr14 injection solution against the comparators dexamethasone and placebo, respectively:
	• Change from baseline in abduction-rotation pain VAS for active external rotation
Logistic regression model with treatment group, center and baseline value (positive/negative) as qualitative factors	Applicable for analyses at visits 5 and 7 for both treatment comparisons Tr14 injection solution against the comparators dexamethasone and placebo, respectively:
	• Jobe
	• Painful arc
Cochran-Mantel-Haenszel (CMH) test for ordered categorical responses stratified by center	Applicable for analyses at visits 5 and 7 for both treatment comparisons Tr14 injection solution against the comparators dexamethasone and placebo, respectively:
	• Patient’s global assessment
	• Investigator’s global assessment

ANCOVA = analysis of covariance; DASH = disability of arm, shoulder, hand; ROM = range of motion; Tr14 = Traumeel; VAS = visual analog scale.

### Sample size calculation

Estimation of sample size is based on the primary efficacy variable, change from baseline in abduction rotation pain VAS for active external rotation. A one-sided *t*-test of non-inferiority at level 0.025 based on a non-inferiority margin of 13 mm and a standard deviation of 25 mm for the response variables achieves a power of 80% computed for equal treatment effects if the sample size is set to 60 patients per active treatment group in the per protocol set. Assuming a dropout rate of 6.25%, at least 160 patients should be randomized (i.e. 64 patients per active treatment group and 32 in the placebo group).

## Discussion

The corticosteroids most commonly used in clinical trials for problems with the rotator cuff are methylprednisolone acetate and triamcinolone acetonide [8]. However, as crystalline corticosteroids, the appearance of these products in the vial is considerably different to Tr14 injection solution. Dexamethasone was chosen as the comparator corticosteroid as visually the solution is similar to Tr14 injection solution, thus aiding blinding of those administering the injections. The dose of dexamethasone was chosen to provide equivalence to 40 mg methylprednisolone acetate or 40 mg triamcinolone acetonide [20]. This dose of dexamethasone also provided a 2 ml injection, which was the same as the quantity of Tr14 injection solution to be used, again assisting blinding. A once-weekly dosing interval was chosen as an appropriate time interval to allow comparison with other injection therapies. A total of three injections was chosen as, in clinical practice, if there are no signs of improvement after 3 weeks of injections, it is unlikely that treatment would be continued.

To the best of our knowledge, dexamethasone has only been investigated in shoulder injuries on two previous occasions, first by Plafki *et al.* [21] using 4 mg dexamethasone-21-palmitat (equivalent to 2.5 mg dexamethasone) injected into the subacromial bursa once with ultrasound guidance for the treatment of subacromial impingement. This study was hindered by the need to stop the local anesthetic only arm of the study due to pain aggravation in some patients rendering the arm ethically unacceptable. However, results in both dexamethasone and triamcinolone acetate (10 mg) treatment groups were similar, suggesting that dexamethasone can provide similar results to triamcinolone acetate. The authors conclude that subacromial steroid injections could prevent the need for surgery in at least half of patients with chronic subacromial impingement syndrome.

The second study by Shibata *et al.* [22] was conducted in patients with full thickness rotator cuff tear, which was a specific exclusion criterion for our study, so the results, although positive, are not of relevance to our study population.

There has been some concern that subacromial corticosteroid injections could be detrimental to the recovery of the rotator cuff tendon. Animal studies have shown detrimental effects of repeated corticosteroid injections on the rotator cuff of rats. Consequent damage to the ultrastructure of collagen molecules has been shown experimentally to weaken collagen fibers and precipitate rotator cuff injuries, with authors suggesting that this could translate into humans, so caution should be exercised [23-25]. However, a study by Bhatia *et al.* [26] suggests that corticosteroid use in patients with subacromial impingement should not be considered a causative factor in rotator cuff tears. This retrospective, case-controlled study compared patients with subacromial impingement syndrome according to the number of subacromial corticosteroid injections they had received (less than three versus three or more). Analysis by magnetic resonance imaging (MRI) showed no significant difference between the two groups in the incidence of rotator cuff tear (p < 1.0).

These concerns serve to highlight the potential need for an effective and safe treatment for rotator cuff syndrome that is not associated with potential detrimental effects on the rotator cuff tendon. In a previous non-randomized, observational study, Tr14 injection solution injections were found to be non-inferior to NSAID injections (mainly diclofenac) for the treatment of epicondylitis [15]. Although the study was designed to demonstrate non-inferiority, markedly greater improvements in pain at rest, change in extensional joint mobility and change in torsional joint mobility were observed, along with greater satisfaction and better tolerability reports from patients for Tr14 injection solution versus NSAID injections. This current study has been designed to investigate whether Tr14 injection solution could provide an effective alternative to corticosteroids with the potential for a better safety profile. This could expand the range of treatments available to clinicians for the treatment of rotator cuff syndrome, providing greater patient choice.

The American Academy of Orthopaedic Surgeons (AAOS) guidelines on ‘Optimizing the Management of Rotator Cuff Problems’ state that they cannot recommend for or against the use of subacromial corticosteroid injections in the treatment of rotator-cuff-related symptoms in the absence of full thickness tear [27]. This is due to a lack of compelling evidence resulting in an unclear balance between benefits and potential harm. There is even less evidence about the efficacy of natural medications. It is hoped that the results of this trial will assist in providing more evidence to support physicians in their management of rotator cuff disease. Investigation of the efficacy and place in therapy of Tr14 injection solution is ongoing with further randomized-controlled trials underway.

## Abbreviations

AAOS: American academy of orthopaedic surgeons
AE: Adverse event
ANCOVA: Analysis of covariance
CMH: Cochran-mantel-haenszel
COX: Cyclo-oxygenase
COX-2: Cyclo-oxygenase type 2
DASH: Disability of arm: shoulder: hand
EU: European union
GCP: Good clinical practice
GMP: Good manufacturing practice
HbA_1c_: Glycosylated fraction of hemoglobin
ICH: International conference on harmonization
IUD: Intrauterine device
LOCF: Last observation carried forward
MedDRA: Medical dictionary for regulatory activities
MRI: Magnetic resonance imaging
NSAID: Non-steroidal anti-inflammatory drug
ROM: Range of motion
TENS: Transcutaneous electrical nerve stimulation
Tr14: Traumeel
VAS: Visual analog scale
WHO: World Health Organization
WMA: World medical assembly
